# A Novel Manifold Dual-Microchannel Flow Field Structure with High-Performance Heat Dissipation

**DOI:** 10.3390/mi13091420

**Published:** 2022-08-28

**Authors:** Xing Yang, Kabin Lin, Daxing Zhang, Shaoyi Liu, Baoqing Han, Zhihai Wang, Kunpeng Yu, Wenzhi Wu, Dongming Ge, Congsi Wang

**Affiliations:** 1 Key Laboratory of Electronic Equipment Structure Design, Ministry of Education, Xidian University, Xi’an 710071, China; 2 Guangzhou Institute of Technology, Xidian University, Guangzhou 510555, China; 3 CETC No. 38 Research Institute, Hefei 230088, China; 4 Beijing Institute of Spacecraft System Engineering, Beijing 100094, China

**Keywords:** electronics cooling, manifold microchannel, double-microchannel, numerical simulation, thermal performance

## Abstract

With the development of miniaturization and integration of electronic devices, the conventional manifold microchannels (MMCs) structure has been unable to meet the heat dissipation requirements caused by the rapid growth of internal heat flux. There is an urgent need to design a new heat dissipation structure with higher heat dissipation capacity to ensure the working stability and life of electronic devices. In this paper, we designed a novel manifold dual-microchannel (MDMC) cooling system that embedded the microchannel structure into the manifold microchannel structure. The MDMC not only has good heat dissipation performance that can meet the development needs of electronic equipment to miniaturization and integration, but also has a compact structure that does not increase the overall thickness and volume compared with MMC. The high temperature uniformity and heat transfer performance of MDMC are significantly improved compared to MMC. The *T*_max_ is reduced by 13.6% and 17.5% at the heat flux density of 300 W/cm^2^ and 700 W/cm^2^, respectively. In addition, the influence of the inlet−2 velocity and the total microchannels number on the heat transfer performance of the MDMC structure are numerically investigated. The results show that the decrease rate of *T*_max_ and Δ*T* is about 6.69% and 16% with the increase of inlet−2 velocity from 1.2 m/s to 2.4 m/s and microchannels number from 10 to 48, respectively. At the same time, the best temperature uniformity is obtained when the number of microchannels is 16.

## 1. Introduction

According to statistics, the reliability of the system will decrease by 50% when the temperature of electronic devices increases by 10 °C. The cooling system can reduce the temperature of electronic devices and maintain the stability of electrical performance, which has become an indispensable part of electronic device design [1,2]. With the development of miniaturization and integration of electronic devices, the internal heat flux density is increasing, which puts forward higher requirements for the heat dissipation capacity of cooling technology. However, the conventional cooling technology finds it difficult to meet such enormous heat dissipation demand [3,4]. Tuckerman and Pease [5] in 1981 firstly proposed the concept of a parallel microchannel heat sink, which has high heat dissipation specific surface area and heat dissipation efficiency compared with the traditional large channel. It has become one of the most promising technologies to solve thermal management problems in various electronics with high power density, and has been successfully applied to electronic chips and laser devices. After that, a large number of scholars proposed many improvement methods, such as turbulence structure [6,7], secondary flow [8,9], pulse flow [10,11], nanofluid [12,13], surface modification [14], and rough surface [15,16], from the directions of discussing heat exchange law [17,18], improving heat dissipation efficiency [19,20], improving flow field and temperature uniformity [21,22], and reducing pressure drop [23,24].

Harpole and Eninger [25] in 1991 firstly proposed the manifold microchannel (MMC) heat sink structure, which added a manifold system to the traditional microchannel (TMC) structure. It could greatly improve temperature uniformity and pressure drop compared to traditional microchannels because the original long straight microchannels are divided into short and curved microchannel units [26,27,28]. Kim et al. [29] experimentally studied an air-cooling MMC structure. Compared with the TMC structure, this structure achieved about 35% reduction in thermal resistance. The CFD simulation results of Zajac [30] also showed that the MMC structures could reduce the temperature by about 50% compared to the TMC structure at the same pressure drop. These studies confirmed that the MMC structure has broad prospects in the field of high-density electronic heat dissipation. Escher et al. [31,32] studied the influence of geometric structure parameters on the heat transfer performance, and preliminarily determined the optimal geometric design size, which provided a reference for the actual processing and application of the MMC structures. Subsequently, Everhart et al. [33] firstly proposed a simple MMC structure packaging scheme with high repetition rate, and applied it to SiC power devices. The experimental results showed that the thermal resistance is less than 0.1 °C/W when the heat flow is 600 W/cm^2^. For the optimization of the MMC structure, Tang et al. [34] designed a tapered inlet manifold channel structure, which further improved the uniformity of the fluid velocity distribution of the MMC structure. Yang et al. [35] designed a better pressure drop characteristics structure, which was combined with the secondary inclined channel. The research results published in “Nature” by Erp et al. [36] showed a processing technology that directly etched microchannels on the silicon surface of the chip and realized direct contact heat transfer between the coolant and the hot spots on the chip surface, which greatly reduced the thermal resistance along the way and further improved the heat transfer performance of the MMC structure.

Although much work has been carried out on the MMC structure, a single fluid channel form cannot meet the rapid growth of heat generation with the continuous increasing power density in various electronic components, which is expected to be more than 1000 W/cm^2^ in the future [35]. Therefore, making the cooling system achieve higher heat dissipation in a smaller volume space has become the key to the design of the cooling system. At the same time, the microchannel system is prone to leakage, blockage, and other problems compared to conventional channels [37,38]. Once this problem occurs in a single microchannel, it will directly lead to the collapse of the cooling system, while multi-microchannels can reduce the possibility of the collapse of the cooling system to a greater extent. Therefore, the optimized structure of the heat sink should not only meet the heat dissipation requirements of high-power devices, but also meet the design requirements of microchannels. The heat transfer performance of the heat sink is different under different flow distributions, and the flow distribution of fluids is affected by the geometry structure and the fluid properties. Acrivos et al. [39] modified the Bernoulli equation by adding a modified momentum term to determine the flow distribution in the channel, and pointed out that the forces such as friction and inertia in the flow interact with the reduction of the channel size, which can lead to the change of the flow distribution. Subsequently, Kim et al. [40] also confirmed that the flow distribution is significantly related to header shape and Reynolds number. Therefore, exploring the heat transfer performance of heat sink at different structural sizes and fluid properties can guide the design and optimization of heat sink structures.

In this paper, we designed a novel manifold dual-microchannel (MDMC) heat sink structure by embedding the microchannel structure into the manifold microchannel structure. Compared with the MMC structure, the MDMC structure does not increase the thickness and volume of the overall structure, which can meet the development needs of electronic equipment in miniaturization and integration. More importantly, the MDMC structure further improves the temperature uniformity and heat transfer performance over the MMC structure. At the same time, the influence of inlet−2 velocity and the total microchannel number on the heat dissipation capability of the MDMC structure are analyzed and explained. The results show that the cooling system can obtain better heat transfer performance by controlling the microchannels number in the range of 12–16 and selecting a smaller inlet−2 velocity under meeting the actual cooling requirements. These provide a reference for the design and application of the cooling system in the high heat flux density device.

## 2. Numerical Simulation of the MDMC Heat Sink

### 2.1. Geometrical Model of the MDMC Heat Sink

**Figure 1:**
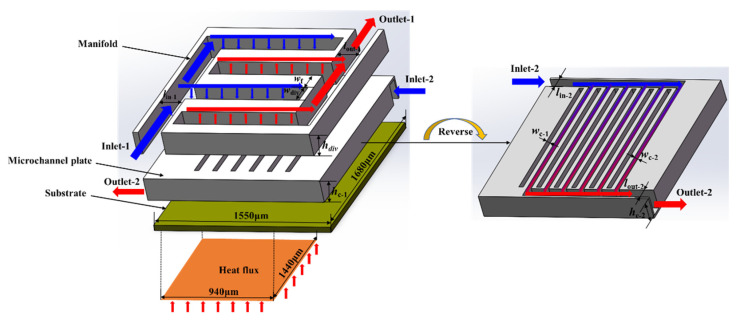
The schematic diagram of the MDMC heat sink.

Figure 1 is a schematic diagram of the MDMC heat sink concept. The whole structure can be divided into three layers: the upper layer is the coolant distribution manifold structure, the middle layer is the microchannel plate, including two microchannel structures, and the lower layer is the substrate, including the heating surface. Compared with the MMC heat sink, the MDMC structure adds an internal microchannel on the microchannel plate to maximize the heat dissipation capacity. The internal and external microchannels of the MDMC structure adopt a cross-arrangement layout to ensure the temperature uniformity of the heat dissipation device. In addition, other structures remain unchanged to take advantage of the MMC structure itself. Table 1 presents the sizes of the MDMC structure and the variation range in the simulation analysis. The size of the heating surface is 1400 × 940 μm^2^. Silicon is chosen as the structure material and water is the coolant. In the optimization work, the structure pressure drop and temperature change under different microchannel widths are simulated and analyzed to achieve the best heat transfer performance of the MDMC structure.

### 2.2. Governing Equations and Boundary Conditions

In this study, the computational fluid dynamics software ANSYS FLUENT 2021 R1 is used to solve the flow and heat transfer of the MDMC based on a three-dimensional fluid–solid coupling model, and the coupled pressure–velocity heat transfer problem is solved by using the finite volume method and coupling algorithm. The momentum and energy equations are discretized using a second-order upwind scheme. For continuity, the velocity calculation results are considered to converge to residuals less than 10^−6^ and the residuals of the energy equation are less than 10^−9^. The main assumptions are as follows:

1. The flow of the two flow fields do not interfere with each other, and there is no connected area inside, maintaining absolute independence.
2. The flow in the two flow fields are single phase, laminar, and incompressible.
3. Thermophysical properties are constant for both fluid and solid heat sink.
4. Gravitational effects, viscous dissipation, and heat loss to the environment are ignored.

Based on the above assumptions, the governing equations of the fluid in the two flow fields include the continuity equation, the momentum equation, and the energy equation. The continuity equation can be expressed as

(1) ∇⋅u=0

The momentum equation can be expressed as

(2) $$\left(u\cdot \nabla \right)\rho_{f}u=-\nabla p+\mu \nabla^{2}u$$

The energy equation can be expressed as

(3) $$\rho_{f}c_{p,f}\left(u\cdot \nabla T\right)=k_{f}\nabla^{2}T$$

The energy equation for the solid domain can be expressed as

(4) $$k_{s}\nabla^{2}T=0$$

where *u* is the velocity of the fluid (m/s); *ρ_f_* is the density of the fluid (kg/m^3^); *μ* is the dynamic viscosity of the fluid (Pa s); *c_p,f_* is the specific heat capacity of the fluid (J/(kg⋅°C)); *k_f_* is the thermal conductivity of the fluid (W/(m⋅°C)); *k_s_* is the thermal conductivity of the solid (W/(m⋅°C)).

The inlets of the two flow fields are both set as velocity-inlet, where the initial inlet temperature is 20 °C, and different inlet velocity values are set to study the effect on heat dissipation performance. The outlets of the two flow fields are both set to pressure-outlet equal to 0. The solid–liquid interface satisfies the conditions of temperature consistency, heat flux continuity, and no-slip boundary layer. The heating surface is applied uniform heat loads with different heat flux values to study the effect on heat dissipation performance. Meanwhile, other external boundary surfaces are set to insulated. The specific values of boundary conditions are shown in Table 2.

### 2.3. Heat Transfer Performance Evaluation Parameters Calculation

In order to evaluate the heat transfer performance of the heat sink structure, the maximum temperature and temperature difference of the heating surface, and the pressure drop between the inlet and outlet are used as evaluation parameters. The meaning and calculation equations of the corresponding parameters are specifically expressed in this section.

The temperature uniformity is represented by the difference between the maximum temperature and the minimum temperature of the heating surface; it can be expressed as

(5) $$\Delta T=T_{\max}-T_{\min}$$

where *T*_max_ and *T*_min_ are the maximum and minimum temperature of the heating surface (°C).

The pressure drop between the inlet and outlet is used to represent the pressure loss during fluid flow, and the pressure drop in the two flow fields can be expressed as

(6) $$\Delta P_{1}=P_{\mathrm{in}-1}-P_{\mathrm{out}-1}$$

(7) $$\Delta P_{2}=P_{\mathrm{in}-2}-P_{\mathrm{out}-2}$$

where *P*_in−1_ and *P*_out−1_ are the inlet and outlet pressures of the flow field−1 (Pa); *P*_in−2_ and *P*_out−2_ are the inlet and outlet pressures of the flow field−2 (Pa).

### 2.4. Mesh Independence and Simulation Verification

**Figure 2:**
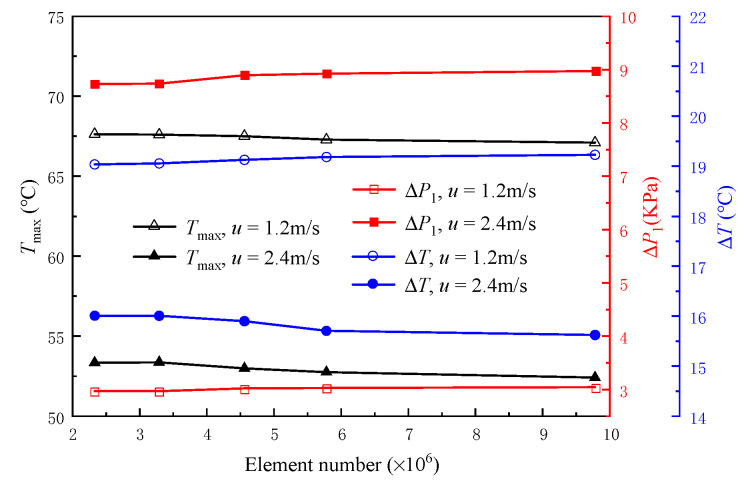
The *T*_max_ and Δ*P*_1_ of the MDMC structure with different meshes.

To verify the mesh independence, five groups of different numbers of meshes were set, which were 2.33 × 10^6^, 3.29 × 10^6^, 4.58 × 10^6^, 5.78 × 10^6^, and 9.81 × 10^6^, respectively, to investigate the difference in thermal simulation results of MDMC structures. The inlet−1 velocity is kept equal to the inlet−2 velocity, the inlet velocities of 1.2 m/s and 2.4 m/s are taken as the verification conditions, and *T*_max_, Δ*T*, and Δ*P*_1_ are selected as the evaluation parameters, as shown in Figure 2. It can be seen that the change values of the three evaluation parameters are lower than 0.6% when the number of meshes increases from 5.78 × 10^6^ to 9.81 × 10^6^. In order to save computational time, the mesh with the number of about 5.78 × 10^6^ is selected for all numerical simulations.

**Figure 3:**
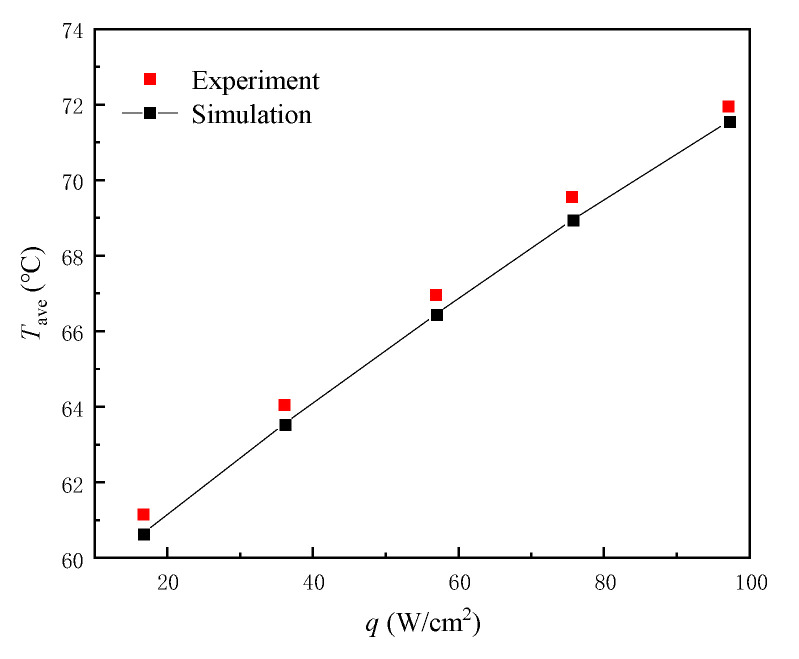
Comparison of average temperature between simulation and experiment.

In order to verify the accuracy of the simulation results, we establish the same MMC structure basic unit model of the experiment [41,42] and select some of these working conditions for simulation. The simulation and experimental results of the average temperature of the heating surface are compared, as shown in Figure 3. The maximum difference of average temperature between the simulation and the experimental results is 0.6 °C, which is smaller than the experimental error of temperature (±1 °C) indicated in the study. At the same time, the size and simulation processes of the MMC and MDMC structures are similar to some extent, which indirectly guarantees the accuracy of the MDMC structure simulation in this work.

## 3. Simulation Results and Discussion of the MDMC Heat Sink

### 3.1. The Heat Transfer Performance Comparison of MMC and MDMC

**Figure 4:**
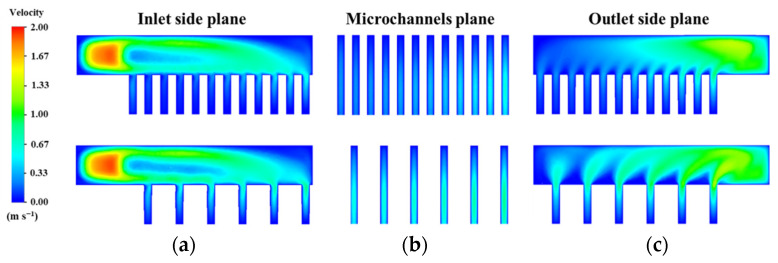
The fluid velocity distribution between MMC and MMDC structures. (**a**) Inlet side plane; (**b**) microchannels plane; (**c**) outlet side plane.

**Figure 5:**
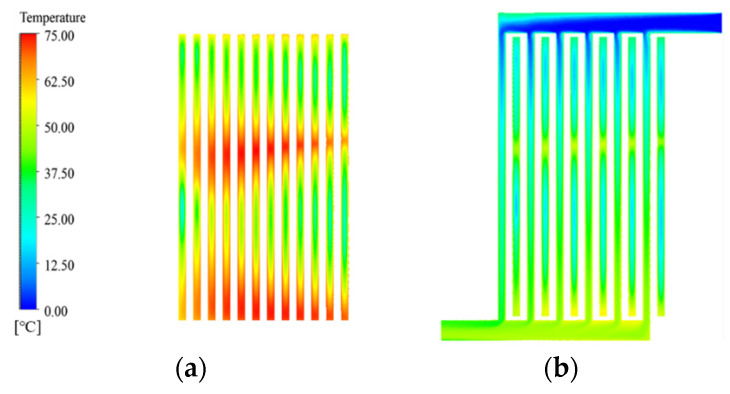
The temperature distribution of microchannel of the MMC and MMDC structures. (**a**) MMC; (**b**) MDMC.

**Figure 6:**
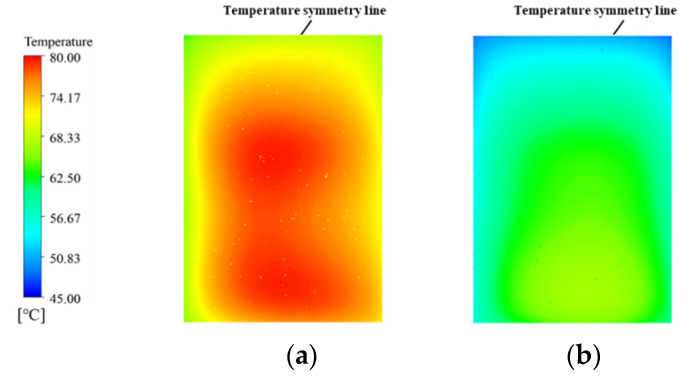
The temperature distribution of heating surface of the MMC and MMDC structures. (**a**) MMC; (**b**) MDMC.

A major feature of the MDMC is that it transforms half of the microchannels belonging to the MMC (flow field−1 of the MDMC) into a new microchannel named flow field−2. According to the different flow fields, the microchannel region of the MDMC structure is divided into microchannel region of flow field−1 (MRFF−1) and microchannel region of flow field 2 (MRFF−2). The fluid velocity and temperature distribution in the microchannels of the MMC and MDMC were compared at the same total number of microchannels (*N* = 12) and initial conditions (*u*_in−1_ = *u*_in−2_ = 1.2 m/s, *q* = 600 W/cm^2^), as shown in Figure 4 and Figure 5. It can be seen from Figure 4 that the fluid of the MDMC structure shows a faster velocity distribution. This because the smaller the microchannels number is, the more flow allocated to each microchannel. Meanwhile, the distance between microchannels also increases with the decrease of the microchannel number, resulting in less disturbance between microchannels and a more uniform velocity distribution between microchannels. The fluid of the MDMC structure shows a lower temperature distribution in Figure 5. This is because the MDMC contains two flow fields that can cool each other through heat transfer. The temperature distribution of the heating surface can be used to evaluate the heat dissipation capacity of the MDMC and MMC structures, as shown in Figure 6. It can be seen that the heating surface of the MDMC structure shows a lower temperature distribution. This is closely related to the fluid velocity and temperature distribution in Figure 4 and Figure 5. Therefore, it is directly confirmed that adding the flow field−2 can achieve better heat dissipation.

**Figure 7:**
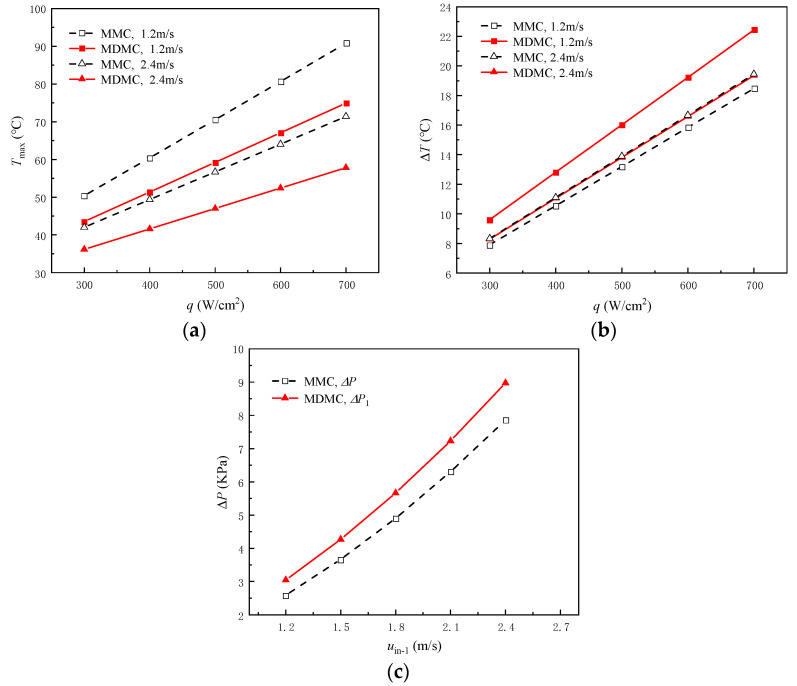
Comparison of heat transfer performance between MMC and MDMC structures. (**a**) *T*_max_; (**b**) Δ*T*; (**c**) Δ*P*.

The heat dissipation capacities of the two structures are compared at different inlet−1 velocity and heat flux density in order to further study the advantages of the MDMC structure. The *T*_max_, Δ*T,* and Δ*P* are used as evaluation parameters, as shown in Figure 7. From Figure 7a, *T*_max_ increases in the two structures with increasing heat flux density. Moreover, the MDMC structure shows lower temperature values than the MMC structure at the same heat flux density. With the heat flux increasing from 300 W/cm^2^ to 700 W/cm^2^, the *T*_max_ decreases by 13.6% to 17.5% when inlet−1 velocity is 1.2 m/s, and by 13.8% to 19% when inlet−1 velocity is 2.4 m/s. This indicates that the MDMC structure can exhibit better heat dissipation capability than the MMC structure at high heat flux density.

Figure 7b shows that the Δ*T* of the MMC increases with increasing inlet−1 velocity, while Δ*T* of the MDMC decreases with increasing inlet−1 velocity at a certain heat flux density. It can be seen that the Δ*T* of the MDMC is about 2.81 °C higher than MMC when inlet−1 velocity is 1.2 m/s, while Δ*T* of the MMC is about 0.07 °C higher than MDMC when inlet−1 velocity is 2.4 m/s. Therefore, the heating surfaces of the two structures show opposite temperature uniformity at different inlet−1 velocity. The MMC structure has only flow field−1, and the temperature uniformity decreases with the increase of inlet−1 velocity. The addition of the flow field−2 causes the *T*_max_ to move from the center left position to the center right position (as shown in Figure 6), which indicates that the temperature distribution of the heating surface for MDMC is affected by the two flow fields. The influence of flow field−2 is greater than that of flow field−1 when inlet−1 velocity is low. Moreover, the influence of the two flow fields is gradually equalized when the inlet−1 velocity increases. Therefore, the temperature uniformity is promoted due to the difference of the flow direction and the inlet direction.

Figure 7c shows that as inlet−1 velocity increases from 1.2 m/s to 2.4 m/s, the Δ*P*_1_ of the MMC structure increases from 2.59 KPa to 7.86 KPa and the Δ*P*_1_ of the MDMC structure increases from 3.04 KPa to 8.97 KPa. This is mainly because the flow area of flow field−1 of the MDMC structure is smaller than that of the MMC structure, but the difference is small. In conclusion, it can be considered that the MDMC structure has better heat transfer performance than MMC.

### 3.2. Influence of Inlet−2 Velocity on Heat Transfer Performance

**Figure 8:**
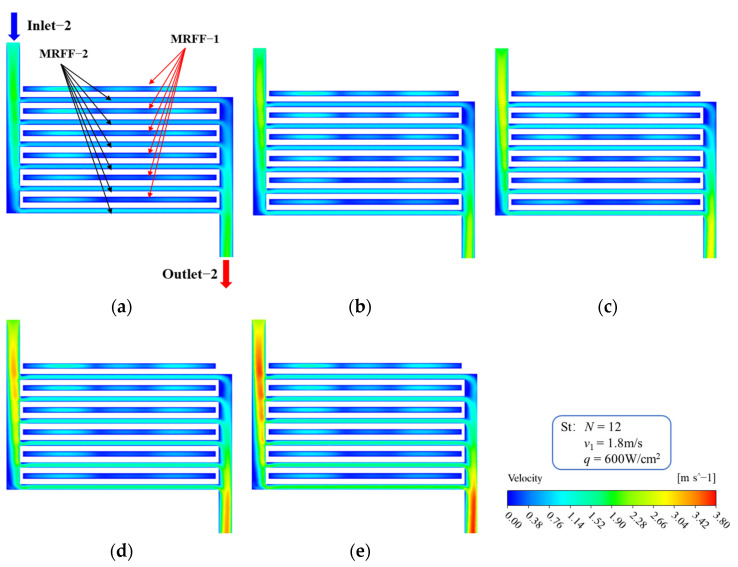
Velocity distribution of microchannel at different inlet−2 velocities. (**a**) *u*_in−2_ = 1.2 m/s; (**b**) *u*_in−2_ = 1.5 m/s; (**c**) *u*_in−2_ = 1.8 m/s; (**d**) *u*_in−2_ = 2.1 m/s; (**e**) *u*_in−2_ = 2.4 m/s.

**Figure 9:**
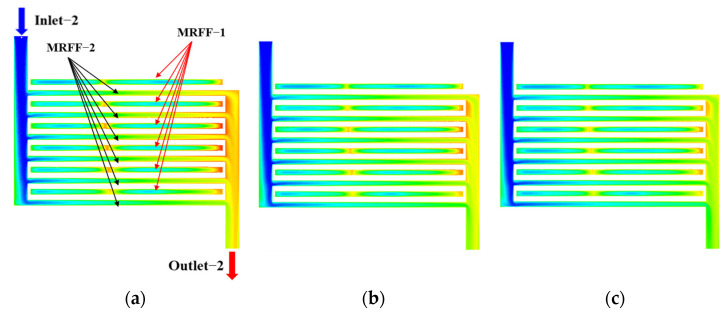

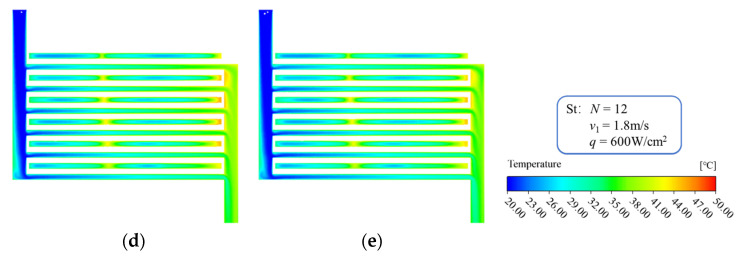
Temperature distribution of microchannel at different inlet−2 velocities. (**a**) *u*_in−2_ = 1.2 m/s; (**b**) *u*_in−2_ = 1.5 m/s; (**c**) *u*_in−2_ = 1.8 m/s; (**d**) *u*_in−2_ = 2.1 m/s; (**e**) *u*_in−2_ = 2.4 m/s.

**Figure 10:**
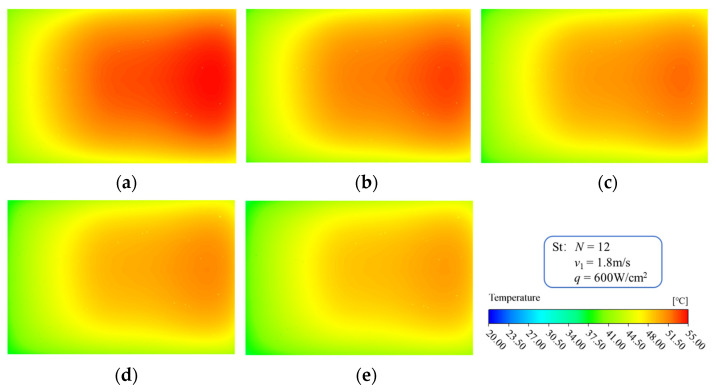
Temperature distribution of heating surface at different inlet−2 velocities. (**a**) *u*_in−2_ = 1.2 m/s; (**b**) *u*_in−2_ = 1.5 m/s; (**c**) *u*_in−2_ = 1.8 m/s; (**d**) *u*_in−2_ = 2.1 m/s; (**e**) *u*_in−2_ = 2.4 m/s.

The fluid flows of the two flow fields of the MDMC structure are independent of each other, but their temperatures affect each other. In this section, the fluid flow characteristics and heat transfer performance at different inlet−2 velocities are analyzed and explained. The initial conditions of Figure 8, Figure 9 and Figure 10 are all set to the heat flux density of 600 W/cm^2^, the inlet−1 velocity of 1.8 m/s, and the microchannels number of 12. Figure 8 shows the fluid velocity distribution of the microchannel region of the MDMC structure at different inlet−2 velocities. For the MREE−2, the fluid velocity of each channel increases with increasing inlet−2 velocity, and the velocity of each channel is different. This is because the distance between each channel and the inlet−2 is different, resulting in a difference in the fluid distribution in each channel. For the MRFF−1, the velocity of each channel remains unchanged at different inlet−2 velocity. This confirms that the fluid flow of the two flow fields will not interfere with each other and will remain absolutely independent.

Figure 9 shows the fluid temperature distribution in the microchannel region of the MDMC structure at different inlet−2 velocities. For MRFF−2, the residence time of the fluid inside the microchannels decreases with increasing inlet−2 velocity. This leads to temperature difference between the inlet and outlet becoming smaller, making the temperature distribution of the flow field more uniform. For MRFF−1, the fluid temperature of each channel decreases with increasing inlet−2 velocity at the same initial conditions. This confirms that MRFF−2 has a certain promoting effect on the heat dissipation performance of MRFF-1, and improves with the increase in inlet−2 velocity.

Figure 10 shows the temperature distribution of the heating surface of the MDMC structure at different inlet−2 velocities, which is a direct reflection of the fluid flow and temperature distribution laws. From the perspective of the global temperature distribution, the inlet−2 is located in the upper left part, and the outlet−2 is located in the lower right part of the heating surface. Therefore, the maximum temperature of the heating surface is located in the center of the right side and gradually decreases along the left direction. From the perspective of temperature distribution change, the inlet−2 velocity has a certain effect on temperature distribution of the heating surface. On the one hand, the temperature of the heating surface decreases with increasing of inlet−2 velocity, which is mainly because it improves the convective heat transfer capacity of the fluid. On the other hand, the temperature uniformity of the heating surface increases with increasing inlet−2 velocity. However, the temperature uniformity of the heating surface in the MDMC structure is simultaneously affected by the two flow fields, and the improvement of the temperature uniformity is small.

**Figure 11:**
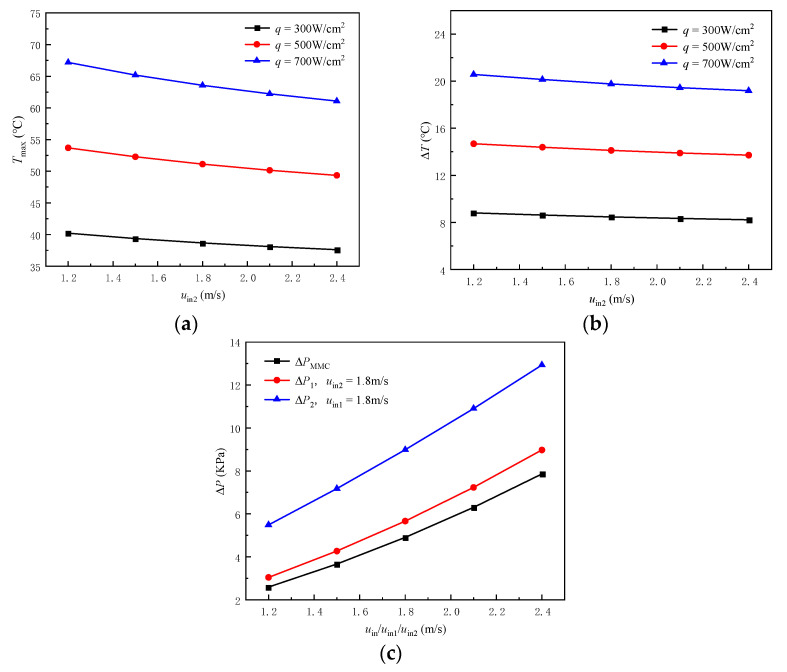
Heat transfer performance of MDMC at different inlet velocities. (**a**) *T*_max_; (**b**) Δ*T*; (**c**) Δ*P*.

In order to further study the influence of inlet−2 velocity on heat dissipation performance, the *T*_max_, Δ*T*, and Δ*P* variations with inlet−2 velocity are shown in Figure 11. The *T*_max_ decreases with increasing inlet−2 velocity, and shows a greater decrease rate at high flux density. It can be seen that when the heat flux density of the heating surface is 700 W/cm^2^, the *T*_max_ decreases from 67.19 °C to 61.08 °C as inlet−2 velocity increases from 1.2 m/s to 2.4 m/s, and the decrease rate is 9.09%. Compared with the heat flux of 300 W/cm^2^, the decrease rate increases by 2.6%, and compared with the heat flux of 500 W/cm^2^, the decrease rate increases by 0.99%. This is because a single flow field can fully meet the heat dissipation requirements at low heat flux. At the same time, the microchannel fluid temperature is low, and the temperature coupling between the two flow fields is not obvious. The decrease rate of the Δ*T* is around 6.69% as inlet−2 velocity increases from 1.2 m/s to 2.4 m/s, which is independent of the heat flux density. There is a linear relationship between Δ*P* and inlet velocity, and it increases with the increase of inlet velocity. Moreover, the Δ*P*_2_ is larger than the Δ*P*_1_ at the same inlet velocity, which indicates that it is more likely to choose a smaller inlet−2 velocity in meeting the actual application requirements.

### 3.3. Influence of Microchannel Number on Heat Transfer Performance

**Figure 12:**
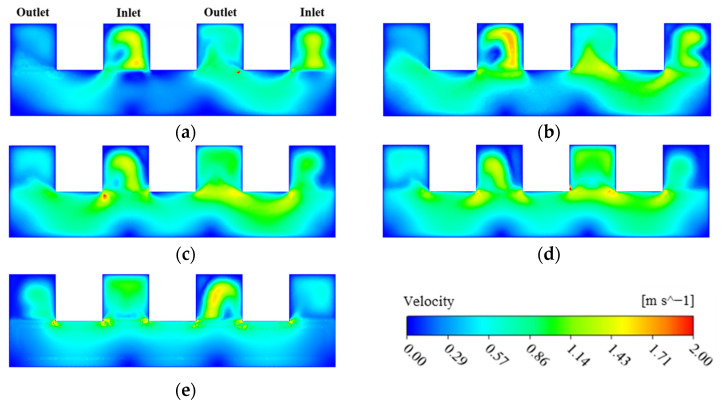
The velocity distribution of the channel under the flow field-1. (**a**) *N* = 10; (**b**) *N* = 12; (**c**) *N* = 16; (**d**) *N* = 24; (**e**) *N* = 48.

**Figure 13:**
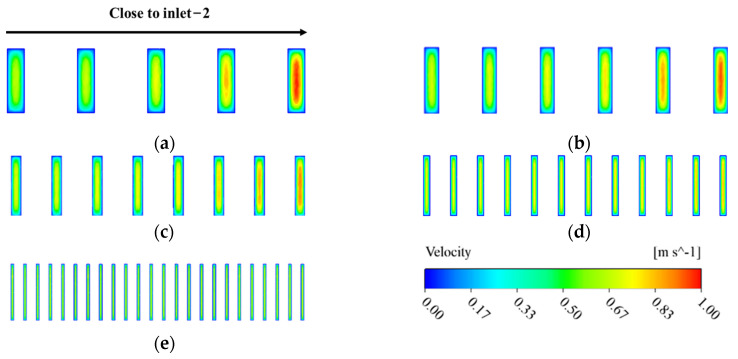
The velocity distribution of the channel section of the flow field−2. (**a**) *N* = 10; (**b**) *N* = 12; (**c**) *N* = 16; (**d**) *N* = 24; (**e**) *N* = 48.

**Figure 14:**
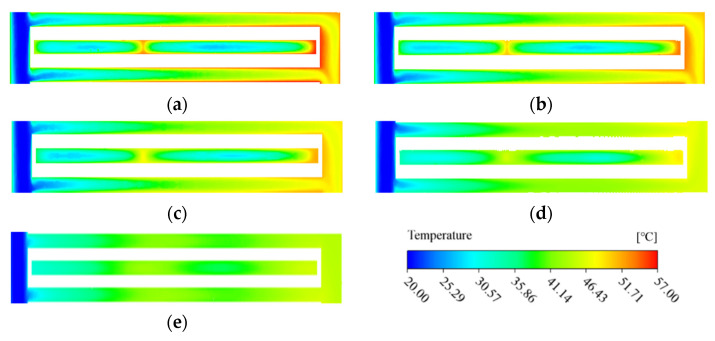
The temperature distribution of microchannel region at different microchannel number. (**a**) *N* = 10; (**b**) *N* = 12; (**c**) *N* = 16; (**d**) *N* = 24; (**e**) *N* = 48.

**Figure 15:**
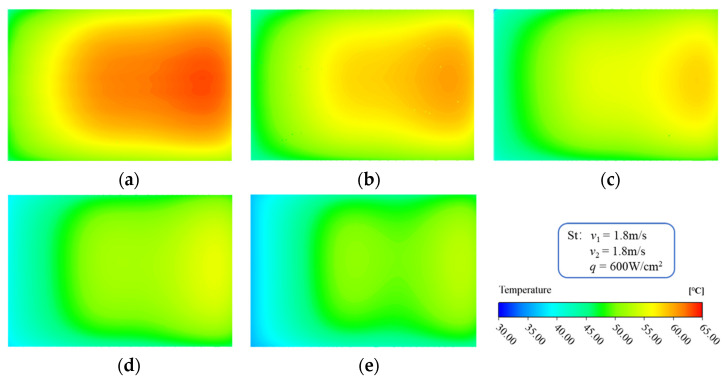
The temperature distribution of heating surface at different microchannel number. (**a**) *N* = 10; (**b**) *N* = 12; (**c**) *N* = 16; (**d**) *N* = 24; (**e**) *N* = 48.

The fluid flow behavior of the two flow fields of the MDMC structure is affected by the microchannels number. This section analyzes and explains the fluid flow characteristics and heat transfer performance at different microchannel numbers. The initial conditions of Figure 12, Figure 13, Figure 14 and Figure 15 are all set to the heat flux density of 600 W/cm^2^, the inlet−1 velocity of 1.8 m/s, and the inlet−2 velocity of 1.8 m/s. Figure 12 shows the velocity distribution of the channel section of the flow field-1 at the different microchannel numbers. The channel fluid first flows into each microchannel from each inlet manifold, then impinges on the bottom of the microchannel to reverse the direction of the fluid flow, and finally flows into each outlet manifold. The microchannel width decreases with increasing of the microchannels number, resulting in greater fluid pressure in the process of entering and leaving the microchannel, which improves the convective heat dissipation capacity of the fluid. Meanwhile, the aspect ratio of the microchannel increases with increasing of the microchannels number. An excessively large aspect ratio may cause the fluid to not reach the bottom of the microchannel when the flow direction is reversed in the microchannel, which reduces the flow velocity near the bottom of the microchannel and then reduces the convection heat dissipation capacity of the fluid. According to the fluid velocity distribution, the microchannels number between 12 and 24 may be more conducive to the heat dissipation of the flow field−1 of the MDMC structure.

Figure 13 shows the velocity distribution of the channel section of the flow field−2 at different microchannel numbers. It can be seen that the fluid distribution in the channel is related to the distance between the channel and the inlet−2. The closer to the inlet−2, the lower the fluid velocity in the channel, and the difference gradually decreases with increasing of the microchannels number. The fluid will be pressurized to produce a jet phenomenon when entering the flow field from the inlet, so that the channels away from the inlet can distribute more fluid. Meanwhile, the microchannel width decreases with increasing of the microchannels number, increasing the pressure of fluid from the inlet channel to each microchannel, improving the fluid uniformity of velocity distribution in each microchannel. For each single microchannel, the fluid flow near the wall is restricted due to channel wall frictional resistance. At the same time, the fluid velocity of a single microchannel decreases with increasing microchannel width. The decrease of inlet velocity can weaken the frictional resistance of the wall, and further improves the uniformity of the fluid velocity.

The flow behavior of the fluid determines the heat dissipation capability of the cooling system. Figure 14 shows the temperature distribution of the microchannel region in the MDMC structure. For a single flow field, the specific area of heat dissipation increases with the increase of the microchannels number, which improves the cooling efficiency of the structure. The fluid temperature difference between the flow field−1 and −2 decreases with increasing of the microchannels number, which is mainly due to the shortening of the heat conduction distance between microchannels. Figure 15 shows the temperature distribution of the heating surface. The high-temperature region of the heating surface is close to the outlet−2 and the overall temperature decreases with increasing the microchannels number, which is consistent with the temperature distribution of the fluid in the microchannel region.

**Figure 16:**
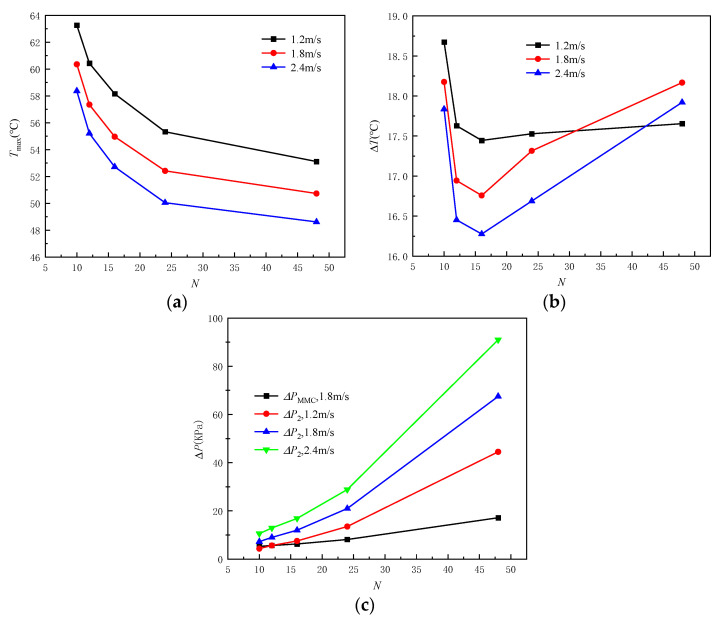
Heat transfer performance of the MDMC at different microchannels number. (**a**) *T*_max_; (**b**) Δ*T*; (**c**) Δ*P*.

In order to further study the influence of microchannels number on heat dissipation performance, the *T*_max_, Δ*T,* and Δ*P* variations with the microchannels number are shown in Figure 16. The *T*_max_ decreases with increasing of the microchannels number, and the decrease rate is independent of the inlet−2 velocity. It can be seen that the decrease rate is about 16% as the microchannels number increases from 10 to 48. Meanwhile, the slopes of the three curves gradually decrease with the increase of the microchannels number. It confirms that this is limited to decrease the overall temperature of the heating surface by increasing the microchannels number. The Δ*T* shows the lowest value when the microchannels number is equal to 16, which means that it has the best temperature distribution at this time. When the microchannels number increases from 10 to 16, the Δ*T* decreases, and the decrease rate is independent of the inlet−2 velocity. However, when the microchannels number exceeds 16, the Δ*T* curve has an inflection point and begins to rise gradually, and shows a faster rising rate at the larger inlet−2 velocity. This is because increasing the microchannels number not only improves the flow uniformity and shortens the heat transfer distance, but also increases the aspect ratio of the microchannels and decreases the flow velocity near the bottom of the microchannel. The Δ*P* increases with the increase of the microchannels number, and its increasing rate increases with the increase of the inlet−2 velocity. Considering the changes in *T*_max_, Δ*T,* and Δ*P* varying with the microchannels number, a better heat dissipation effect and power consumption can be obtained when the microchannels number is in the range of 12–16.

## 4. Conclusions

In this work, a manifold dual-microchannel (MDMC) heat sink structure is proposed, which embeds the microchannel structure into the manifold microchannel (MMC) structure. The heat transfer performance of MDMC is studied. The influence of inlet−2 velocity and microchannels number on flow and heat transfer characteristics of the MDMC structure are analyzed and explained. The main points can be summarized as follows::

1. The MDMC structure shows better heat transfer performance than the MMC structure at high flux density and large inlet−1 velocity due to the dual-microchannel flow field. With the heat flux increasing from 300 W/cm^2^ to 700 Wcm^2^, the *T*_max_ decreases by 13.6% to 17.5% when inlet−1 velocity is 1.2 m/s, and by 13.8% to 19% when inlet−1 velocity is 2.4 m/s.
2. The *T*_max_ and Δ*T* decrease with increasing inlet−2 velocity. The *T*_max_ decreases from 67.19 °C to 61.08 °C as inlet−2 velocity increases from 1.2 m/s to 2.4 m/s, and the decrease rate is 9.09%. Compared with the heat flux of 300 W/cm^2^, the decrease rate increased by 2.6%. The Δ*T* decrease rate is around 6.69% as inlet−2 velocity increases from 1.2 m/s to 2.4 m/s, which is independent of the heat flux density.
3. The decrease rate of the *T*_max_ is about 16% as microchannels number increases from 10 to 48, and the decrease rate is independent of the inlet−2 velocity. The best temperature uniformity is obtained when the number of microchannels is equal to 16. When the microchannels number increases from 10 to 16, the Δ*T* decreases, and the decrease rate is independent of the inlet−2 velocity. However, when the microchannels number exceeds 16, the Δ*T* curve has an inflection point and begins to rise gradually, and shows a faster rising rate at the larger inlet−2 velocity.

## Figures and Tables

**Table 1:** The summary of the MDMC structure dimensions.

Parameter	Variable	Dimension (μm)
Length of inlet−1	*l*_in−1_	200
Length of outlet−1	*l*_out−1_	200
Length of inlet−2	*l*_in−2_	100
Length of outlet−2	*l*_out−2_	100
Width of divider	*w*_div_	200
Height of divider	*h*_div_	200
Fin width	*w*_f_	200
Microchannel width of flow field−1	*w*_c−1_	10, 20, 30, 40, 50
Microchannel height of flow field−1	*h*_c−1_	200
Microchannel width of flow field−2	*w*_c−2_	10, 20, 30, 40, 50
Microchannel height of flow field−2	*h*_c−2_	180

**Table 2:** The summary of the MDMC structure boundary conditions.

Parameter	Variable	Dimension
Velocity of inlet−1 (m/s)	*u*_in−1_	1.2, 1.5, 1.8, 2.1, 2.4
Pressure of outlet−1 (Pa)	*P*_out−1_	0
Velocity of inlet−2 (m/s)	*u*_in−2_	1.2, 1.5, 1.8, 2.1, 2.4
Pressure of outlet−2 (Pa)	*P*_out−2_	0
Heat flux (W/cm^2^)	*q*	300, 400, 500, 600, 700

## Data Availability

Not applicable.

## Nomenclature

TMC	traditional microchannel
MMC	manifold microchannel
MDMC	manifold dual-microchannel
MRFF−1	microchannel region of flow field−1
MRFF−2	microchannel region of flow field−2
*c_p,f_*	specific heat capacity of the fluid (J/(kg⋅°C))
*h*	height (μm)
*k*	thermal conductivity (W/(m⋅°C))
*l*	length (μm)
*N*	microchannel number
*P*	Pressure (Pa)
*q*	heat flux of heating surface (W/cm^2^)
*T*	temperature (°C)
Δ*T*	temperature different (°C)
*u*	velocity (m/s)
*w*	width (μm)
*Subscript*	
1	flow field−1
2	flow field−2
c	microchannel
div	divider
f	fluid
in	inlet
max	maximum
min	minimum
out	outlet
s	solid
*Green letters*	
*μ*	dynamic viscosity (Pa s)
*ρ*	density (kg/m^3^)
